# Lower Vitamin C Levels Are Associated With Less Improvement in Negative Symptoms in Initially Antipsychotic-Naïve Patients With First-Episode Psychosis

**DOI:** 10.1093/ijnp/pyac029

**Published:** 2022-05-09

**Authors:** Anders N Myken, Bjørn H Ebdrup, Mikkel E Sørensen, Brian V Broberg, Martin W Skjerbæk, Birte Y Glenthøj, Jens Lykkesfeldt, Mette Ø Nielsen

**Affiliations:** Center for Neuropsychiatric Schizophrenia Research (CNSR) and Centre for Clinical Intervention and Neuropsychiatric Schizophrenia Research (CINS), Mental Health Centre Glostrup, University of Copenhagen, Denmark; Faculty of health and Medical Science, Department of Clinical Medicine, University of Copenhagen, Denmark; Center for Neuropsychiatric Schizophrenia Research (CNSR) and Centre for Clinical Intervention and Neuropsychiatric Schizophrenia Research (CINS), Mental Health Centre Glostrup, University of Copenhagen, Denmark; Faculty of health and Medical Science, Department of Clinical Medicine, University of Copenhagen, Denmark; Center for Neuropsychiatric Schizophrenia Research (CNSR) and Centre for Clinical Intervention and Neuropsychiatric Schizophrenia Research (CINS), Mental Health Centre Glostrup, University of Copenhagen, Denmark; Center for Neuropsychiatric Schizophrenia Research (CNSR) and Centre for Clinical Intervention and Neuropsychiatric Schizophrenia Research (CINS), Mental Health Centre Glostrup, University of Copenhagen, Denmark; Center for Neuropsychiatric Schizophrenia Research (CNSR) and Centre for Clinical Intervention and Neuropsychiatric Schizophrenia Research (CINS), Mental Health Centre Glostrup, University of Copenhagen, Denmark; Center for Neuropsychiatric Schizophrenia Research (CNSR) and Centre for Clinical Intervention and Neuropsychiatric Schizophrenia Research (CINS), Mental Health Centre Glostrup, University of Copenhagen, Denmark; Faculty of health and Medical Science, Department of Clinical Medicine, University of Copenhagen, Denmark; Faculty of Health and Medical Sciences, Department of Veterinary and Animal Sciences, University of Copenhagen, Denmark (Dr Lykkesfeldt); Center for Neuropsychiatric Schizophrenia Research (CNSR) and Centre for Clinical Intervention and Neuropsychiatric Schizophrenia Research (CINS), Mental Health Centre Glostrup, University of Copenhagen, Denmark; Faculty of health and Medical Science, Department of Clinical Medicine, University of Copenhagen, Denmark

**Keywords:** Vitamin C, negative symptoms, first-episode psychoses

## Abstract

Low levels of vitamin C have been observed in patients with schizophrenia and psychosis, and vitamin C may affect the dopaminergic system. Likewise, antipsychotic medication modulates striatal dopamine D2 receptors. We measured vitamin C levels in 52 patients with first-episode psychoses (24 females, age 23.1 ± 5.2 years) and 57 matched HCs (20 females, age 22.7 ± 4.3 years) before and after 6 weeks where patients received aripiprazole monotherapy (mean dose 10.4 mg ± 4.8 mg). At baseline, patients displayed lower levels of vitamin C (57.4 ± 25.9 µM) than controls (72.7 ± 21.4 µM) (t = 3.4, *P* = .001). Baseline symptoms and vitamin C levels were not correlated. Higher baseline vitamin C levels were associated with more improvement in negative symptoms (n = 39, R^2 ^= 0.20, F = 8.2, *P* = .007), but not with age, sex, or p-aripiprazole. Because negative symptoms are generally considered challenging to alleviate, a potential adjunctive effect of vitamin C on treatment response should be tested in future randomized clinical trials.

## Introduction

Psychotic symptoms often lead to changes in lifestyle and eating habits, such as increased consumption of fat and refined carbohydrates (Saugo et al., 2020), which in turn may result in lower intake of minerals and vitamins (Aucoin et al., 2020). Psychosis is associated with increased dopamine turnover in striatum, where dopamine D2 receptors are mostly expressed and hypofunction of the dopaminergic system in other areas in the brain such as the prefrontal cortex (PFC) (Howes and Kapur, 2009). All marketed antipsychotics target the D2 receptor.

Vitamin C is a hydrophilic carbohydrate abundantly present in fruits and vegetables. Humans are unable to synthesize vitamin C and rely solely on dietary intake. In vivo, vitamin C mainly exists in the reduced form, ascorbic acid, and the oxidized form, dehydroascorbic acid. With a daily intake of 400 mg/d or more, a homeostatic state is reached with maximal steady-state concentrations of approximately 70 to 80 µmol/L (Lykkesfeldt et al., 2014). Vitamin C is an electron donor, and high concentrations of vitamin C in the CNS are required for the synthesis of monoamine neurotransmitters. Specifically, vitamin C is a co-factor in the synthesis of serotonin (Englard and Seifter, 1986) and in the transformation of dopamine to norepinephrine (Harrison and May, 2009). Finally, vitamin C has an antagonistic effect on the dopamine D1 and D2 receptors (Tolbert et al., 1992).

Accumulating evidence suggests an association between a poor diet and several psychiatric conditions (Adan et al., 2019), and patients with first-episode psychoses display several nutritional deficiencies, including vitamin C (Firth et al., 2018). A few studies have reported lower vitamin C levels in medicated patients with schizophrenia (Dadheech et al., 2006; Suprapaneni, 2007). In a preclinical study, the effect of haloperidol, risperidone, and clozapine on apomorphine-induced stereotypical movements in mice was enhanced by vitamin C in a dose-dependent manner (Deshpande et al., 2006). A small double-blind intervention study in schizophrenia patients showed that co-administration of vitamin C resulted in an improved response to antipsychotic medication (Dakhale et al., 2005). Whether vitamin C also potentiates the effect of the newer partial dopamine D2 receptor agonists such as aripiprazole, brexipiprazole, and cariprazine has not been investigated.

In the present longitudinal study, vitamin C levels were assessed in antipsychotic-naïve patients with first-episode psychosis (FEP) and a group of matched healthy controls (HCs). We hypothesized that patients at baseline would exhibit lower concentrations of vitamin C in plasma, which would be associated with increased symptoms. Furthermore, we expected that lower baseline vitamin C status in patients would be associated with a poorer effect of 6 weeks of treatment with a partial dopamine agonist, aripiprazole.

## METHODS

### Participants

The present data were collected between 2014 and 2019 as a part of the PECANS II project, a large multimodal study carried out in accordance with the Declaration of Helsinki and approved by the Committee on Biomedical Research Ethics (H-3-2013-149). All participants provided informed consent. The study design was a prospective 6-week follow-up cohort study, which included a total of 66 antipsychotic-naïve patients with FEP and 58 HC, matched on age, sex, and parental socioeconomic status. Patients were recruited from hospitals and outpatient mental health centers in the capital region in Denmark and were between 18 and 45 years of age; were legally competent; and fulfilled the diagnostic criteria of schizophrenia, persistent delusional disorder, acute and transient psychotic disorders, schizoaffective disorder, or other or unspecified non-organic psychotic disorders according to ICD-10 (or schizophrenia schizophreniform- or schizoaffective disorder according to DSM-IVR). The diagnosis was confirmed by a diagnostic interview (Schedules for Clinical Assessment in Neuropsychiatry) (Wing et al., 1990).

Exclusion criteria for patients were any prior exposure to antipsychotics, daily substance abuse or fulfilling the criteria of ongoing substance dependency according to ICD-10/DSM-IVR, recent treatment with antidepressant medication, patients involuntarily admitted or treated, pregnancy, or severe physical illness. Exclusion criteria for controls were first-degree relatives with psychotic symptoms, substance abuse or positive screening of drugs in urine sample, pregnancy, or severe physical illness.

HCs were recruited using internet advertisement (https://www.forsoegsperson.dk/) and matched on age (±2 years of age), gender, and parental socioeconomic status.

### Assessments

Blood samples were acquired in the morning in fasting state at baseline and at 6 weeks follow-up. Blood tubes were kept on ice and immediately centrifuged for 2 minutes, after which the plasma was acidified by adding 10% meta-phosphoric acid containing 2 mM disodium ethylenediaminetetraacetic acid. After another short centrifugation, the samples were stored at −80°C. Time from phlebotomy to storage in the freezer was registered. Vitamin C and its oxidation ratio have been shown to be stable for at least 5 years under these conditions (Lykkesfeldt, 2012). After study completion, all samples were analyzed by means of high-performance liquid chromatography as described in detail elsewhere (Lykkesfeldt, 2000). Analyses were performed at the Department of Veterinary and Animal Sciences, Faculty of Health and Medical Science, University of Copenhagen, Denmark.

Information about use of cannabis, alcohol, and tobacco during the last month was obtained. Height and weight were measured, and body mass index (BMI) was calculated. After baseline examinations, patients received monotherapy with the antipsychotic compound aripiprazole in individual doses balancing clinical effect and side effects.

Psychopathology in patients was evaluated by trained raters using the Positive and Negative Syndrome Scale (PANSS) (Kay et al., 1987). Percentual change in PANSS was calculated (Obermeier et al., 2011). Depressive symptoms were assessed with the Calgary Depression Scale for Schizophrenia (Addington et al., 1993). Level of function was assessed for all participants using the Personal and Social Performance Scale (Morosini et al., 2000).

### Statistics

Statistical analyses were performed using Statistical Package for the Social Sciences for Windows version 25 (SPSS Inc., Chicago, IL, USA). Normal distribution of the data was tested with Shapiro–Wilks test. Group comparisons were performed with independent sample *t* test or chi-squared test.

Multiple stepwise regression with total vitamin C as the dependent variable and group, age, gender, regular tobacco use (yes/no), BMI, fruit/vegetable consumption (multiple times per day/daily/less than daily), and vitamin C supplements (yes/no) as independent variables was performed. Repeated-measures ANOVA was used to examine the effect of time and group on vitamin C levels, and post hoc analyses included independent and paired *t* tests, which were also used for change in psychopathology and level of function. Relation between baseline vitamin C levels and psychopathology, and relation between p-aripiprazole and change in symptoms were examined with Spearman’s correlation coefficient. Multiple regressions were performed with percentual change in PANSS scores corrected for minimal score as the dependent variable and vitamin C, gender, age, and p-aripiprazole as independent variables.

## RESULTS

### Baseline

Vitamin C was obtained from 52 patients and 57 HCs. The groups were well matched, but patients had fewer years of education and more tobacco use (Table 1).

**Table 1.** Demographic and clinical data

	FEP n = 52	HC n = 57	Statistics
Age, y, mean (SD)	23.0 (±5.2)	22.7(±4.3)	t = 0.5, *P* = .655
Gender (f/m)	24/28	20/37	χ² = 1.4, *P* = .240
BMI	24.9 (±6.3)	23.1 (±2.3)	T = 1,8, *P* = .73
Education, y	11.5 (±2.0)	13.7 (±2.0)	***t*** = **−4,0, *P* < .001**
Regular tobacco use (yes/no)	22/30	12/45	***χ²*** = **5.7, *P* = .017**
Regular alcohol use (yes/no)	38/14	49/8	χ² = 2.8, *P* = .094
Regular cannabis use (yes/no)	6/46	2/55	χ² = 2.6, *P* = .108
Fruit and vegetable intake^*a*^	14/18/18	37/12/8	***χ²* = 14.6, *P* = .001**
Vitamin C supplements^*b*^ (yes/no)	7/45	10/47	χ² = 0.3 *P* = .557
Total vitamin C level, *μ*mol/L	57.4 (±25.9)	72,7 (±21.4)	**t = 3.4, *P* = .001**
Vitamin C below/above median^*c*^	32/20	23/34	***χ²* = 4.8, *P* = .027**
Dehydroscorbic acid, *μ*mol/L	1.27 (±1.2)	0.95 (±1.6)	t = 1.0, *P* = .301
Blood processing time, min	6.9(±3.2)	7.4(±3.2)	t = −1.4, *P* = .162

Bold values indicates significant group difference, *P* < .05.

Abbreviation: BMI, body mass index.

^*a*^Consumptions of fruit and vegetables: (multiple times pr. day/daily/less than daily).

^*b*^Counting multivitamin and/or vitamin C.

^*c*^Median for the whole sample was 69.5 μM.

**Figure 1.**
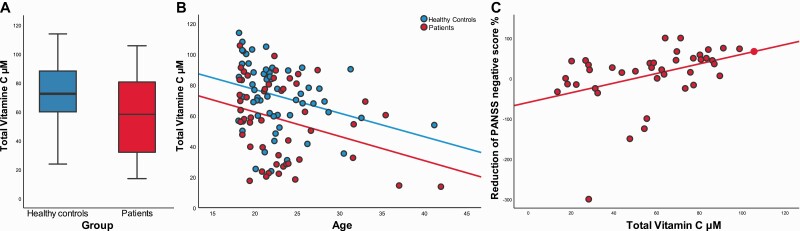
(A) Boxplot of baseline vitamin C divided by group. (B) The relation between age and levels of vitamin C at baseline. (C) Correlation between baseline levels of vitamin C and percentage reduction in negative symptoms corrected for minimum score (baseline − 7) − (follow-up − 7) * 100/(baseline − 7).

At baseline, patients had lower levels of vitamin C levels (t = 3.4, *P* = .001) (Figure 1A). Median vitamin C in the whole sample was 69.5 µM. A higher proportion of patients had vitamin C levels below the median split (62% vs 40% χ² = 4.8, *P* = .027). Multiple stepwise regression with total vitamin C as the dependent variable showed that total vitamin C was associated with group and age (R^2^ = 0.20, F = 12.5, *P* < .001). Group was the main predictor for vitamin C (R^2^ = 0.11, F = 13.2, *P* < .001) (Figure 1B). Variables removed (criteria *P* > .10) were gender (β = .144, t = 1.56, *P* = .12), tobacco use (β = .005, t = 0.55, *P* = .957), BMI (β = .063, t = .69, *P* = .49), and vitamin C supplements (β = .110, t = 1.23, *P* = .22), whereas variable not entered (criteria *P* < .05) was fruit/vegetable consumption (β = .17, t = 1.83, *P* = .07).

Because of group difference in several independent variables (smoking, vitamin C supply, and fruit and vegetable intake), we additionally performed the regression without including group as an independent variable. In this model, vitamin C was associated with age and fruit and vegetable intake (R^2^ = 0.17, F = 10.5, *P* < .001), with age being the main predictor for vitamin C (R^2^ = 0.10, F = 11.6, *P* = .001) when group was excluded. Variables removed (criteria *P* > .10) were gender (β = .145, t = 1.54, *P* = .13), tobacco use (β = .024, t = .26, *P* = .79), BMI (β = .105, t = 1.15, *P* = .25), and vitamin C supplements (β = .09, t = .97, *P* = .33).

Because group was the main predictor, groupwise analyses were performed post hoc and showed that vitamin C levels in HCs were associated with gender and regular tobacco use (R^2 ^=^ ^0.20, F = 6.3, *P* = .003), but only associated with age in patients (R^2^ = 0.10, F = 4.9, *P* = .031). There was no group difference in processing time of the blood samples or in dehydroascorbic acid.

### Follow-up

After 6 weeks, data on 39 patients and 55 HCs were obtained. Drop-out analyses showed no differences between the patients, who completed follow-up examinations and patients, who left the study (all *P* > .1). The patients who remained in the study received a mean dose of 10.3 (range: 2.5–25) mg aripiprazole and had a significant reduction on PANSS-total, -positive, -negative, and -general symptoms. Symptom improvement was not associated with aripiprazole dose or plasma aripiprazole levels. The treatment also resulted in a significant improvement in level of functioning (Table 2).

**Table 2.** Data on patients at baseline and follow up

	Baseline n = 52	6-Week follow-up n = 39	Statistics baseline follow-up
Total vitamin C *μ*mol/L, mean (SD)	57.4 (±25.9)	58.2 (±22.7)	t = 0.7, *P* = .472
PANSS total	75.6 (±14.4)	59.9 (±13.5)	**t = 8.1, *P* < .001**
Positive	18.6 (±4,4)	13.8 (±4.4)	**t = 9.4, *P* < .001**
Negative	19.4 (±5,3)	16.9 (±16.7)	**t = 3.5, *P* = .001**
General	37.6 (±7.5)	29.3 (±7.1,)	**t = 7.7, *P* < .001**
PSP	48.3^*a*^ (±14.2)	56.9^*b*^ (±12.5)	**t = 4,5, *P* < .001**
CDSS	8.3 (±3.9)	4.6 (±4.4)	**t = 4.1, *P* < .001**
BMI	24.9 (±6.6)	24.2(±6.0)	T = 0.8, *P* = .408
Aripiprazole dose (mg/d)		10.3^*c*^ (2.5-25)	
P-aripiprazole (ng/mL)		303.3 (±235.9)	
P-aripiprazole and dehydroaripiprazole (ng/mL)		450.1(±128.8)	

Bold values indicates significant group difference, *P* < .05.

Abbreviations: BMI, body mass index; CDSS, Calgary Depression Scale for Schizophrenia; PANSS, Positive and Negative Symptom Scale; PSP, Personal and Social Performance scale.

^*a*^Consumptions of fruit and vegetables: (multiple times pr. day/daily/less than daily).

^*b*^Counting multivitamin and/or vitamin C.

^*c*^Median for the whole sample was 69.5 µM.

a: n = 51 b: n = 37 c: n = 34

Vitamin C levels were stable over 6 weeks in both patients and controls as a repeated-measure ANOVA showed a main effect of group (F = 13.9, *P* < .001), no effect of time (F = 0.821, *P* = .367), and no group × time interaction (F = 0.081, *P* = .777).

### Vitamin C and Psychopathology

At baseline, PANSS and Calgary Depression Scale for Schizophrenia did not correlate with vitamin C levels (all *P* > .1). Multiple regression showed that improvement in negative symptoms was associated with higher baseline vitamin C (R^2^ = 0.17, F = 8.2, *P* = .007) but not with age, sex, or p-aripiprazole (illustrated as a correlation in Figure 1C). Explorative analyses showed that higher vitamin C was correlated with reductions in emotional withdrawal (N2), poor report (N3), passive-apathetic thinking (N4), and difficulty in abstract thinking (N5) (Spearman’s correlations, all *P* < .01). Analyses using PANSS positive, -general, and -total scores as independent variables were not significant (*P* > .05). Within patients, vitamin C levels did not correlate with aripiprazole dose or p-aripiprazole (*P* > .5).

## Discussion

We found markedly lower vitamin C levels in this sample of antipsychotic-naive patients with FEP, but vitamin C levels did not correlate with severity of symptoms. Levels of vitamin C remained stable during 6 weeks of antipsychotic monotherapy, but symptoms improved during treatment and there was an association between higher level of plasma vitamin C and improvement in negative symptoms.

As hypothesized, our patients exhibited lower vitamin C levels than HCs also after adjusting for dietary habits and substance use. This group difference was present before antipsychotic treatment and was unaffected by aripiprazole, thereby corroborating previous findings (Dadheech et al., 2006; Suprapaneni, 2007). Patients reported less consumption of fruit and vegetables, which was found to be associated with lower baseline vitamin C levels. This indicates that inexpedient dietary habits and poorer nutritional status are present early in the disease and support that metabolic abnormalities are not solely related to antipsychotic treatment (Williamson et al., 2015; Saugo et al., 2020).

Different predictors for vitamin C were observed between the groups; among patients, age was the main explanatory factor, whereas gender and regular tobacco use were explanatory factors in the HC group. The observation that regular tobacco use is associated with lower vitamin C levels is well documented in a normal population (Lykkesfeldt et al., 2000). Because this was not the case in our patients, there may be other disease-related factors contributing to the vitamin C level in patients with psychosis. Nevertheless, we did not, as expected, find any association between vitamin C levels and symptom severity.

In our data, higher vitamin C levels were associated with improvement in negative symptoms. The levels of vitamin C remained stable over time in both groups, which reflected that participants did not undergo any dietary intervention. Primary negative symptoms and cognitive deficits observed in patients with schizophrenia have been linked to dopaminergic hypofunction in the PFC (Collo et al., 2020). Aripiprazole is a partial D_2_-receptor agonist hypothesized to exert an agonistic effect in PFC, where the endogen dopamine levels are reduced (Tuplin and Holahan, 2017), which may in turn improve negative symptoms. The current data could suggest that vitamin C enhances the agonistic effect on D_2_-receptors in PFC. Because aripiprazole also has affinity to other receptor systems, including 5-HT_2A_ receptors (Tuplin and Holahan, 2017), further studies are needed to clarify the possible potentiating effect of vitamin C on negative symptoms.

Finally, it could be speculated that our findings represent the presence of a subgroup of patients with deficit schizophrenia characterized by primary and persistent negative symptoms (Carpenter et al., 1988). Thus, longstanding negative symptoms may underlie inexpedient dietary habits (Kirkpatrick et al., 2006) and in turn partly explain the poorer treatment response on negative symptoms often reported in these patients. Because our sample consisted of acutely ill and newly diagnosed psychotic patients, we were not able to identify a subgroup of patients with deficit syndrome according to the original definition (Carpenter et al., 1988). Future studies on stable patients with and without a high level of primary negative symptoms may be able to explore this.

In conclusion, antipsychotic-naïve patients with FEP exhibited lower vitamin C level than HCs. Although this was not associated with patients’ symptom severity, lower vitamin C levels were associated with more persistent negative symptoms after short-term treatment with a partial D2 agonist. Whether vitamin C potentiates the antipsychotic effect and supplementary vitamin C as adjuvant treatment may improve treatment effect should be further examined in future studies.

## Interest Statement

B.H.E. has received lecture fees and/or is part of the advisory boards of Bristol-Myers Squibb, Eli Lilly and Company, Janssen-Cilag, Otsuka Pharma Scandinavia AB, Takeda Pharmaceutical Company, Boehringer Ingelheim, and Lundbeck Pharma A/S. B.Y.G. is the leader of a Lundbeck Foundation Centre of Excellence for Clinical Intervention and Neuropsychiatric Schizophrenia Research (CINS), which is partially financed by an independent grant from the Lundbeck Foundation based on international review and partially financed by the Mental Health Services in the Capital Region of Denmark, the University of Copenhagen, and other foundations. All grants are the property of the Mental Health Services in the Capital Region of Denmark and administrated by them. She has no other conflicts to disclose. J.L. is Director of the LifePharm Centre for In Vivo Pharmacology, which is partly funded by Novo Nordisk, and has received lecture fees and travel reimbursement from DSM. B.V.B. was employed at CNSR during study design and data collection but is now employed at Novo Nordisk. A.M. received a scholarship from the Lundbeck foundation. M.E.S., M.S., and M.Ø.N. declare no conflicts of interest.
